# Rhizosphere Microenvironments of Eight Common Deciduous Fruit Trees Were Shaped by Microbes in Northern China

**DOI:** 10.3389/fmicb.2018.03147

**Published:** 2018-12-18

**Authors:** Peng Si, Wei Shao, Huili Yu, Xiaojing Yang, Dengtao Gao, Xiansheng Qiao, Zhiqiang Wang, Guoliang Wu

**Affiliations:** ^1^Laboratory of Cultivation Physiology, Zhengzhou Fruit Research Institute, Chinese Academy of Agricultural Sciences, Zhengzhou, China; ^2^College of Forestry, Henan Agricultural University, Zhengzhou, China; ^3^College of Horticulture, Henan Agricultural University, Zhengzhou, China

**Keywords:** deciduous fruit trees, rhizosphere microenvironment, soil physicochemical properties, soil enzyme activity, soil microbial community, redundancy analysis, Pearson correlation

## Abstract

The rhizosphere microenvironment is the site of nutrient circulation and microbial community formation, and thus is an ongoing topic of research. Although research on this topic is extensive, studies into the rhizosphere microenvironment of fruit trees remain rare. To elucidate the mechanisms driving the fruit tree rhizosphere microenvironment, we assessed soil physicochemical properties, enzyme activities, the community-level physiological profile (CLPP) and microbial diversity in rhizospheric soils of eight common deciduous fruit trees in northern China. We found that the available minerals, pH, enzyme activities, microbial utilization of six types of carbon (C) substrates, and microbial diversity in the rhizosphere varied among tree species. Redundancy analysis (RDA) showed that rhizosphere microenvironmental parameters (ammonia nitrogen content, soil pH and invertase activity) were closely related to the soil microbial community. Further analysis revealed that the soil microbial utilization of six C sources, nitrate nitrogen content, and invertase activity were negatively correlated with *Ambiguous* species and *Alternaria*; however, these groups were positively correlated with pH. The ammonia nitrogen content was positively correlated with C source utilization and negatively correlated with *Ambiguous, Lysobacter, Nitrospira, Alternaria, Fusarium*, and *Colletotrichum*. Interestingly, invertase was closely linked to the microbial community, especially fungal diversity, and was positively correlated with plant-beneficial microbes such as *Mortierella, Geomyces, Lysobacter*, and *Chaetomium*, but negatively correlated with pathogenic microbes such as *Alternaria, Fusarium*, and *Colletotrichum*. Hence, rhizosphere soil physicochemical properties, enzyme activities and microbial community were significantly affected by tree species. Additionally, a variety of environmental factors were closely related to the microbial community in the rhizospheric soils of eight species of deciduous fruit trees.

## Introduction

Deciduous fruit trees have been planted north of the Yangtze River in China, including apples, pears, walnuts, peaches, grapes, cherries, apricots, etc. (Tian and Zhang, 2017). The rapid growth of deciduous fruit production in recent years has become an important force driving the growth of fruit crops worldwide (Feng et al., 2014; Srivastava et al., 2015). The rhizosphere, which links root traits to functions, is the site of nutrient acquisition, nutrient cycling and microbial community formation (Mommer et al., 2016; Chen et al., 2017), and affects not only fruit tree growth, but also the yield and quality of fruit (Babu et al., 2015; Mazzola et al., 2015; Soni et al., 2016). Research into the differences and commonalities of the rhizosphere environment among different deciduous fruit trees in northern China may facilitate better understanding of the rhizosphere of different tree species, improve scientific knowledge to inform more effective fertilization methods in the future, and finally, generate new ideas for the development of specialized fruit tree fertilizers. Recently, the Chinese government proposed a policy to reduce the use of pesticides and fertilizers and thus curb the trend of increasing agricultural nonpoint-source pollution. The traditional fertilization method in China has disadvantages, including low efficiency of fertilizer utilization and water waste (Liu et al., 2010; Miao et al., 2011). Therefore, elucidating the mechanisms underlying fruit tree rhizosphere microenvironments is necessary.

The rhizosphere is the narrow zone of soil that surrounds roots and is influenced by plant root exudates (Philippot et al., 2013; Qiu et al., 2014). The chemistry of the rhizosphere can be affected by plant roots in various ways, including through release and uptake of organic compounds, the gas exchange associated with respiration of roots and rhizosphere microorganisms, and root absorption and release of water and nutrients (Neumann and Römheld, 2014). Moreover, the physical properties of rhizosphere soil are modified by root growth through the soil and the presence of polymeric substances (Marschner, 2012; Neumann and Römheld, 2014). The rhizosphere ecosystem of fruit trees is based on a fruit tree-soil-microbe relationship and their interactions with environmental conditions; a harmonious rhizosphere ecosystem developed through fertilization is beneficial for nutrient absorption and normal growth and development of fruit trees (Zhang et al., 2009). Hence, the application of fertilizer and the rhizosphere environment have a close relationship.

The rhizosphere effect states that plants release up to 40% of their photosynthetic products into the rhizosphere (Singh et al., 2017), resulting in a phenomenon in which the rhizosphere microbial population density is much higher than that in the surrounding bulk soil (Bais et al., 2006; Berendsen et al., 2012). In the rhizosphere, 10^11^ microbial cells per gram of root and more than 30,000 prokaryotic species are present, making it one of the most dynamic interfaces on Earth (Egamberdieva et al., 2010; Berendsen et al., 2012; Philippot et al., 2013). According to their effects on plant growth, rhizosphere microbes are divided into beneficial microbes, deleterious rhizosphere microbes, and neutral microbes exerting no direct effects on pathogens or plants (Hu et al., 2004). Plant-beneficial microbes have been widely studied for their positive effects on plant growth and health (Lugtenberg, 2015). They can facilitate nitrogen (N), phosphorus (P), and mineral uptake by the plant, and can also secrete plant hormones into the rhizosphere to promote plant growth (Oberhänsli et al., 1991; Bottini et al., 2004). These microbes can produce secondary metabolites such as HCN, 2,4-diacetylphloroglucinol and pyoluteorin to antagonize pathogenic fungi, thereby indirectly promoting plant growth (Defago et al., 1990). In addition, they can directly or indirectly participate in the auxin pathway and modulate the plant root architecture, in turn affecting the absorption of nutrients during beneficial plant-microorganism interactions (Sukumar et al., 2013). Thus, plant-beneficial microbes might also increase soil nutrient uptake, reducing the need for fertilizers and preventing the accumulation of nitrates and phosphates in agricultural soils (Yang et al., 2009; Hongoh and Ohkuma, 2011). Deleterious microbes cause major yield reductions in economic crops (Oerke et al., 1994). In particular, plant pathogenic fungi are a more significant problem than plant pathogenic bacteria in temperate climates (Mendes et al., 2013). In the rhizosphere, a fierce battle is fought between plant-beneficial microbes and deleterious microbes (Berendsen et al., 2012). Hence, the composition of the microbial community in the rhizosphere plays a crucial role in the function of plants through influences on their physiology and development (Mendes et al., 2013).

Here, we compared the rhizosphere soil in terms of physicochemical properties, enzyme activity, community-level physiological profile (CLPP) and microbial structure diversity among eight deciduous fruit trees that are common in northern China, and analyzed the relationship between rhizosphere microbes and microenvironmental factors.

## Materials and Methods

### Site Description

The experiment was performed at Xinxiang Integrated Test Base, Zhengzhou Fruit Research Institute, Chinese Academy of Agricultural Sciences (35°7′40″N, 113°45′57″E), located in the Henan Province of China. The average elevation of the study area was 80 m above sea level. The climate was a typical temperate continental monsoon climate with a mean annual temperature of 14°C. Mean annual precipitation was ~573.4 mm. The topsoil contained 125.89 mg/kg available P, 202.6 mg/kg available potassium (K), 1.39 mg/kg ammonia nitrogen, and 5.62 mg/kg nitrate nitrogen, with a pH-H_2_O of 8.05.

### Experimental Design

In March 2012, deciduous fruit trees of eight species were planted in northern China: apples (strain: Huashuo; rootstock: *Malus robusta* Rehd.), pears (strain: Wanqiuhuangli; rootstock: *Pyrus betulaefolia* Bge.), apricots (strain: Katy apricot; rootstock: self-rooted), cherries (strain: Longguan; rootstock: ZY-1), peaches, (strain: Zhongyou 4; rootstock: *Prunus davidiana*), grapes (strain: Kyoho grape; rootstock: self-rooted), walnuts (strain: Zhonghe 1; rootstock: self-rooted) and pomegranates (strain: Tunisia; rootstock: self-rooted). A plot that was not planted with fruit trees was used as a control (CK). Nine trees (plant spacing: 3 m × 3 m) were planted in each plot (plot area: 36 m^2^). A randomized complete block design was utilized with three replications. To avoid interference, each blank plot was situated between two adjacent plots. A total of 375 kg/ha of compound fertilizer (N, P_2_O_5_, and K_2_O, each at 15%) and 9,000 kg/ha of organic fertilizer was applied every September to all plots. No other chemicals were used in any of the plots.

### Soil Sampling

Rhizosphere soil samples were collected in May 2017. The deciduous layer was first removed. Then, the rhizosphere soil 10–20 cm above the ground was collected, placed in a sterile bag, and stored on ice. The soil samples were passed through a 2-mm aperture sterile screen. Within each plot, three trees were randomly selected for rhizosphere soil collection, and three rhizospheric soils were mixed into one soil sample. Thus, each treatment includes three replicates. All samples were subsequently stored at −80°C.

### Measurement of Soil Physicochemical Properties

The pH was measured using a pH meter (PHS-3C; Leici, Shanghai, China) at a 1:5 (w/v) ratio of soil to distilled water (Lu, 2000). The soil organic matter (SOM) content was determined using a colorimetric method involving oxidation with potassium dichromate (Nelson and Sommers, 1996). Nitrate (${\text{NO}}_{3}^{-}$-N) and ammonium (${\text{NH}}_{4}^{+}$-N) were extracted using 1.0 M KCl at a 1:10 soil-to-solution ratio. After shaking for 1 h, the extracts were filtered and then measured using an Automated Discrete Analyzer (CleverChem 380‘DeChem-Tech Inc., Hamburg, Germany) (Lu, 2000). The available K was extracted in 1 M ammonium acetate using flame photometry [FP6431; INASA Inc., Shizuoka, Japan (Lu, 2000)]. The available P was extracted from the soil sample with 0.5 M NaHCO_3_ (pH 8.5) and measured spectrophotometrically (Tu-1901; Persee Inc., Beijing, China) as blue molybdate-phosphate complexes formed through partial reduction with ascorbic acid (Lu, 2000).

### Soil Enzyme Activities

According to the method described by Yao and Huang (2006), the invertase activity was measured. Briefly, 2 g of fresh soil was incubated with 15 mL of 8% sucrose, 5 mL of phosphate buffer (pH 5.5), and five drops of methylbenzene at 37°C for 24 h, and the reducing sugars released were then analyzed at 508 nm using a spectrophotometer (Yu et al., 2016) (Tu-1901; Persee Inc.). Urease activity was determined as described by Yao and Huang (2006). Five grams of fresh soil was incubated with 1 mL of methylbenzene, 10% urea and 20 mL of citrate buffer (pH 6.7) at 37°C for 24 h. Then, the content of p-nitrophenol was measured at 578 nm using a spectrophotometer (Yu et al., 2016) (Tu-1901; Persee Inc.). Catalase activity was measured according to Li et al. (2008) by incubating 2 g of fresh soil sample with 40 mL of distilled water and 5 mL of 0.3% H_2_O_2_ at 120 rpm for 20 min, and then 25 mL of the filtrate was titrated with KMnO_4_ after adding 5 mL of 1.5 mol/L H_2_SO_4_ to terminate the reaction (Yu et al., 2016). The alkaline phosphatase activity was measured according to the method described by Guan (1986), by incubating 2 g of fresh soil with 20 mL of borate saline buffer (pH 9.6) and five drops of methylbenzene at 37°C for 24 h. The content of p-nitrophenol in the filtrate was determined using a spectrophotometer at 660 nm (Yu et al., 2016) (Tu-1901; Persee Inc.). The β-glucosidase activity was measured according to the method described previously by Eivazi and Tabatabai (1988). Briefly, 1 g of fresh soil was incubated with 4 mL of phosphate buffer (pH = 6.0) and 1 mL of substrate (p-nitrophenyl-β-d-glucopyranoside) at 37°C for 1 h. Then, 1 mL of 0.5-mol/L CaCl_2_ and 4 mL of Tris buffer (pH 12) were added to stop the reaction, and the filtrate was measured using a spectrophotometer at 400 nm (Yu et al., 2016) (Tu-1901; Persee Inc.). Cellulase activity was estimated as described by Schinner and Von Mersi (1990). Briefly, 1 g of fresh soil was incubated with 10 mL of 2 M acetate buffer (pH 5.5) containing carboxymethyl cellulose sodium salt (0.7%) for 24 h at 50°C. Then, the filtrates of the soil-substrate slurries were subjected to a color reaction, and the Prussian blue produced was analyzed colorimetrically in a microplate reader at 690 nm (Bio-Rad, Hercules, CA, USA).

### Analysis of the Community-Level Physiological Profile (CLPP)

A CLPP was constructed using the Biolog EcoPlate (Biolog Inc., Hayward, CA, USA). Each Biolog EcoPlate contained 31 carbon (C) sources in triplicate as well as three negative controls in a 96-well-plate format (Montes-Borrego et al., 2013). Briefly, 1 g of soil and 99 ml of 0.85% sterilized NaCl solution were added to an autoclaved triangular flask, and the flask was shaken at 120 rpm for 30 min and then stored at 4°C for 30 min. A total of 150 μL of the resulting solution was placed in each well, and cultivated at 25°C for 192 h. Then, the plates were read every 24 h by the Biolog MicroStation™ reader at both 590 and 750 nm (Guanghua et al., 2008) (Biolog Inc.). Biolog EcoPlate substrates were divided into six groups based on C sources (PM, polymers; CH, carbohydrate; PC, phenolic compounds; CA, carboxylic acids; AA, amino acids; AN, amines) (Choi and Dobbs, 1999; Rutgers et al., 2016; Kolton et al., 2017). The 96-h data, which were collected in the exponential phase, were used for constructing the CLPPs of deciduous fruit trees. Principal component analysis (PCA) was used to assess tree species-related differences in CLPPs after normalization of the absorbance associated with each substrate (Kolton et al., 2017), and the utilization of the six C source groups was calculated to assess tree species-specific catabolic activity (Jiang et al., 2013; Wu et al., 2013).

### DNA Extraction and Polymerase Chain Reaction (PCR)

The total genomic DNA was extracted from each soil sample using TIANamp Soil DNA Kit 107 (Tiangen Biotech Inc., Beijing, China) following the manufacturer's instructions. The quality and integrity of the DNA were assessed based on its A260/280 ratio and agarose gel electrophoresis. The DNA extracted from three independent soil samples served as a template to amplify the 16S rRNA gene and the internal transcribed spacer (ITS) region. The bacterial 16S rRNA gene was amplified with the universal primer pair B341F (5′-CCTACGGGNGGCWGCAG-3′) and B785R (5′-GACTACHVGGGTATCTAATCC-3′) (Klindworth et al., 2013). The fungal-specific primers, ITS3F (5′-GATGAAGAACGYAGYRAA-3′) and ITS4R (5′-TCCTCCGCYYATTGATATGC-3′), were employed to amplify the fungal ITS region (Toju et al., 2012). PCR amplification was performed using the KAPA HiFiHot Start ReadyMix PCR Kit in a GeneAmp PCR System 9700 instrument (Life Technologies, Carlsbad, CA, USA). The PCR reactions were conducted with 25-μL total volume reaction cocktails consisting of 12.5 μL of KAPA HiFi HotStart ReadyMix (2×), 0.25 μmol L^−1^ of each primer and 10 ng of the DNA template. Amplification was performed with the following thermal profile: 3 min of initial denaturation at 95°C followed by 25 cycles of denaturation at 95°C for 30 s, annealing at 55°C for 30 s, extension at 72°C for 30 s, and then a final extension at 72°C for 5 min. After purification, the PCR products were quantified using 2100 Bioanalyzer System (Agilent Inc., Santa Clara, CA, USA) (Mueller et al., 2000) and pooled at equal concentrations. Then, amplicon sequencing was performed using a MiSeq platform (Illumina, San Diego, CA, USA) at Genewiz, Inc. (Suzhou, China).

### Data Analysis

The 16S rRNA and ITS data were analyzed using the QIIME (Quantitative Insights Into Microbial Ecology; ver. 1.9.1) data analysis package (Li et al., 2017; Qiao et al., 2018; Zhang et al., 2018). Operational taxonomic units (OTUs) at 97% similarity were used to perform rarefaction analysis, and to calculate the richness and diversity indexes. Details of the sequencing data are shown in Tables S1, S2. Sample normalization was conducted through rarefaction. The Chao and Shannon indices were calculated as measures of microbial richness and diversity. Microbial parameter data were analyzed using one-way analysis of variance with tree species as the factor. The values were considered significantly different at a 95% confidence level. Values shown in figures and tables correspond to the average of triplicate data ± standard error (SE). At the phylum level, the relative abundances of 7 major bacterial phyla (>1.0% of total composition in all samples) and 4 major fungal phyla (>0.01% of total composition in all samples) were shown using one-way analysis of variance with tree species as the factor. Principal coordinates analysis (PCoA) was performed using the OTU data to identify differences in microbial communities between the control and treatment soils. The heatmap figures, PCoA and Venn diagrams were produced using R Package. The PCA was performed using Canoco 4.5 Microcomputer Power, Ithaca, NY, USA).

### Data Availability

The sequence reads generated in this project are available online (http://www.ncbi.nlm.nih.gov/sra) under accession number SRP161632.

## Results

### Rhizosphere Soil Physicochemical Properties and Enzyme Activities

The available nutrient contents and pH of the rhizosphere soil differed significantly among the eight deciduous fruit tree species sampled at the initial fruiting stage in northern China (Table 1 and Table S3). The rhizosphere soil available mineral contents (except available P, calcium and magnesium) were obviously greater, while the pH was significantly lower, for the deciduous fruit trees compared to the control samples. A detailed comparison among trees showed that the walnut and pomegranate soil pH levels were lower than those of other deciduous fruit trees. Among the fruit trees, the apple soil had the highest ammonia nitrogen content and the lowest nitrate nitrogen content, while the pear soil showed the opposite pattern. The available K content of peach soil was much higher than of the other samples, while the lowest available K content was found in the grape and cherry soils. The available P content of the walnut soil was significantly higher than that of the other fruit trees, while the grape, pomegranate, apricot, cherry, and peach soils had markedly less available P. The organic matter (OM) content of the walnut soil was greatest, while that of apple was lowest. The available zinc and copper contents of walnut rhizosphere soil were highest, while those of apricot soil were lowest. The available content of trace elements in the cherry rhizosphere soil was generally low. The iron and calcium contents of apple and pear rhizospheric soils were higher than the others. Among these fruit trees, the available boron content of peach was the highest, and grape had the highest available magnesium. Hence, significant differences were observed in rhizosphere soil available nutrient contents and pH among fruit trees at the initial fruiting stage.

**Table 1:** Rhizosphere soil physicochemical properties of different deciduous fruit trees in northern China.

	**pH**	**NH_**4**_-N** **(mg/kg)**	**NO_**3**_-N** **(mg/kg)**	**Available K** **(mg/kg)**	**Available P** **(mg/kg)**	**Organic matter** **(g/kg)**
CK	8.05 ± 0.03*a*	1.39 ± 0.42*f*	5.62 ± 0.2*d*	202.6 ± 1.16*f*	125.89 ± 0.85*c*	16.31 ± 0.36*e*
Grape	7.25 ± 0.03*e*	2.23 ± 0.13*ef*	6.63 ± 0.67*cd*	231.2 ± 5.65*ef*	21.33 ± 0.65*e*	19.55 ± 0.07*d*
Walnut	6.95 ± 0.03*g*	5.71 ± 0.26*ab*	6.73 ± 0.62*cd*	356.67 ± 3.39*c*	296.1 ± 10.11*a*	32.91 ± 0.38*a*
Pomegranate	7 ± 0.06*g*	4.9 ± 0.56*bc*	10.37 ± 0.14*b*	454.37 ± 8.04*b*	34.85 ± 1.15*de*	22.78 ± 0.25*c*
Apricot	7.8 ± 0*b*	3.23 ± 0.12*de*	7.83 ± 0.04*c*	282.93 ± 2.36*d*	29.12 ± 1.52*de*	23.13 ± 0.12*c*
Cherry	7.3 ± 0*de*	3.73 ± 0.21*d*	6.33 ± 0.04*cd*	240.4 ± 0.86*e*	35.62 ± 1.88*de*	24.61 ± 0.03*b*
Peach	7.35 ± 0.03*d*	4.27 ± 0.59*cd*	10.09 ± 0.6*b*	555.62 ± 31.23*a*	40.58 ± 1.48*d*	19.9 ± 0.18*d*
Apple	7.15 ± 0.03*f*	6.16 ± 0.4*a*	6.01 ± 1.02*d*	483.83 ± 4.97*b*	119.61 ± 8.45*c*	16.73 ± 0.12*e*
Pear	7.7 ± 0*c*	1.91 ± 0.27*f*	12.68 ± 0.26*a*	323.53 ± 2.11*c*	214.45 ± 1.17*b*	23.06 ± 0.3*c*

*Values are means ± SE. Suffixes in the same row indicate statistical significance at p < 0.05 using one-way analysis of variance with tree species as the factor*.

Furthermore, the activity of various soil enzymes was markedly affected by the fruit tree species (Table 2). Soil urease, a key enzyme involved in soil N cycling, can catalyze the hydrolysis of urea into ammonia. Among the fruit trees, the urease activity in the walnut soil was significantly higher than that in the others; the peach and apple soils had the lowest urease activities. There were no significant differences among the soils of the grapes, apricots, and cherries. Soil alkaline phosphatase hydrolyzes organic P in the soil into available P, which can be used by the crop. In the fruit tree rhizosphere, the alkaline phosphatase activity of apricot was significantly higher than that of the other species, while grape, peach, and apple exhibited relatively low alkaline phosphatase activity. Catalase promotes the decomposition of hydrogen peroxide into water and oxygen and can reduce the toxic effects of hydrogen peroxide on plants. Sucrase is related to multiple soil factors, including SOM, N, and P content, microbial abundance, and soil respiration rate. Catalase and sucrase activities were highest in the pomegranate soil, while the soils from the apple subfamily (apple and pear) had the lowest catalase and sucrose activities. Cellulase helps to degrade cellulose and is an important enzyme in the C cycle. Among all trees tested, the cellulase activity was highest in the soil from the grape tree. Interestingly, the cellulase activities in soils of all Rosaceae crops were significantly lower than those in other soils. β-glucosidase is an essential enzyme in the C cycle, which degrades cellobiose, cellotriose and other low-molecular-weight dextrins into glucose. The β-glucosidase activity of the apricot soil was significantly higher than those of other species, while it was significantly lower in the soils from the grape and apple subfamily (apple and pear).

**Table 2:** Soil enzyme activities of different deciduous fruit trees in northern China.

	**Urease** **NH3-N mg/(g·24 h)**	**Alkaline phosphatase** **Phenol mg/(g·24 h)**	**Catalase** **H_**2**_O_**2**_ mg/(g·20 min)**	**Invertase** **glucose mg/(g·24 h)**	**Cellulase** **glucose mg/(g·h)**	**β-glucosidase** **P-nitrophen mmg/(kg·h)**
CK	0.53 ± 0.02*cd*	0.49 ± 0*f*	1.68 ± 0.02*c*	3.59 ± 0.2*f*	2.08 ± 0.02*c*	33.65 ± 2.54*d*
Grape	0.53 ± 0.04*cd*	0.78 ± 0.03*e*	1.76 ± 0.01*b*	7.05 ± 0.14*d*	2.54 ± 0.08*a*	32.91 ± 1.3*d*
Walnut	0.61 ± 0.03*a*	1.53 ± 0.04*ab*	1.78 ± 0*ab*	8.84 ± 0.17*c*	2.36 ± 0.07*b*	133.68 ± 10.45*c*
Pomegranate	0.59 ± 0.01*ab*	1.43 ± 0.05*b*	1.81 ± 0.02*a*	13.62 ± 0.28*a*	2.24 ± 0.02*b*	123.07 ± 12.34*c*
Apricot	0.54 ± 0*bcd*	1.6 ± 0.09*a*	1.77 ± 0.01*b*	10.46 ± 0.15*b*	1.51 ± 0.03*ef*	286.96 ± 23.96*a*
Cherry	0.48 ± 0.05*de*	1.21 ± 0.06*c*	1.77 ± 0.01*b*	8.94 ± 0.24*c*	1.4 ± 0.03*fg*	127.63 ± 7.23*c*
Peach	0.46 ± 0.03*e*	0.76 ± 0*e*	1.69 ± 0.01*c*	7.05 ± 0.06*d*	1.68 ± 0.02*d*	181.19 ± 11.48*b*
Apple	0.46 ± 0.04*e*	1.05 ± 0.03*d*	1.58 ± 0*e*	6.7 ± 0.2*de*	1.31 ± 0.02*g*	41.78 ± 2.63*d*
Pear	0.56 ± 0.02*abc*	1.53 ± 0.05*ab*	1.62 ± 0.01*d*	6.21 ± 0.06*e*	1.62 ± 0.03*de*	42.78 ± 2.13*d*

*Values are means ± SE. Suffixes in the same row indicate statistical significance at p < 0.05 using one-way analysis of variance with tree species as the factor*.

### Rhizosphere Soil Microbial Community-Level Physiological Profiles

**Figure 1:**
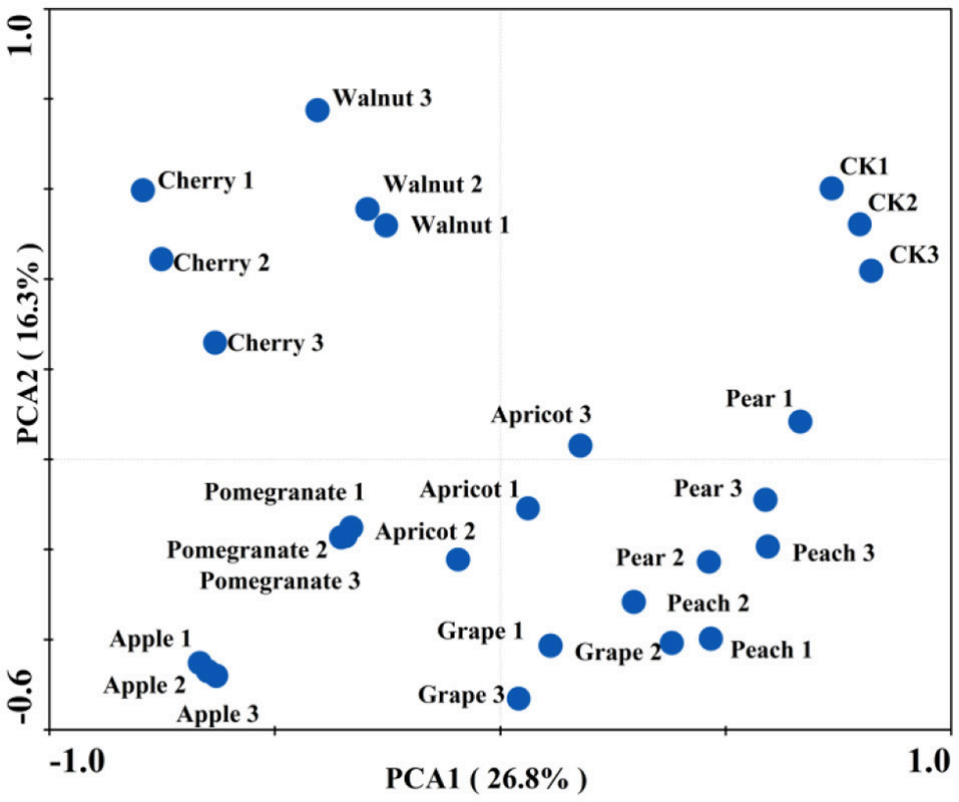
Effect of different deciduous fruit trees on the root-associated microbial community carbon source utilization in northern China. Principal component analysis (PCA) of carbon substrate utilization patterns obtained with Biolog Eco-plates, showing different deciduous fruit trees.

The PCA of the soil microbial CLPP showed that the various deciduous fruit trees affected the functional structure of the soil microbial community (Figure 1). Two PCAs accounted for 43.1% of the total variation and each soil sample formed its own cluster. The control clusters exhibited positive trends for PCA1 and PCA2, while the apple and pomegranate clusters were both negatively correlated with PCA1 and PCA2. Moreover, PCA analysis showed three relatively distinct groups, with the control cluster on the right side, the cherry-walnut cluster on the left side, and the other fruit trees clustered below.

**Figure 2:**
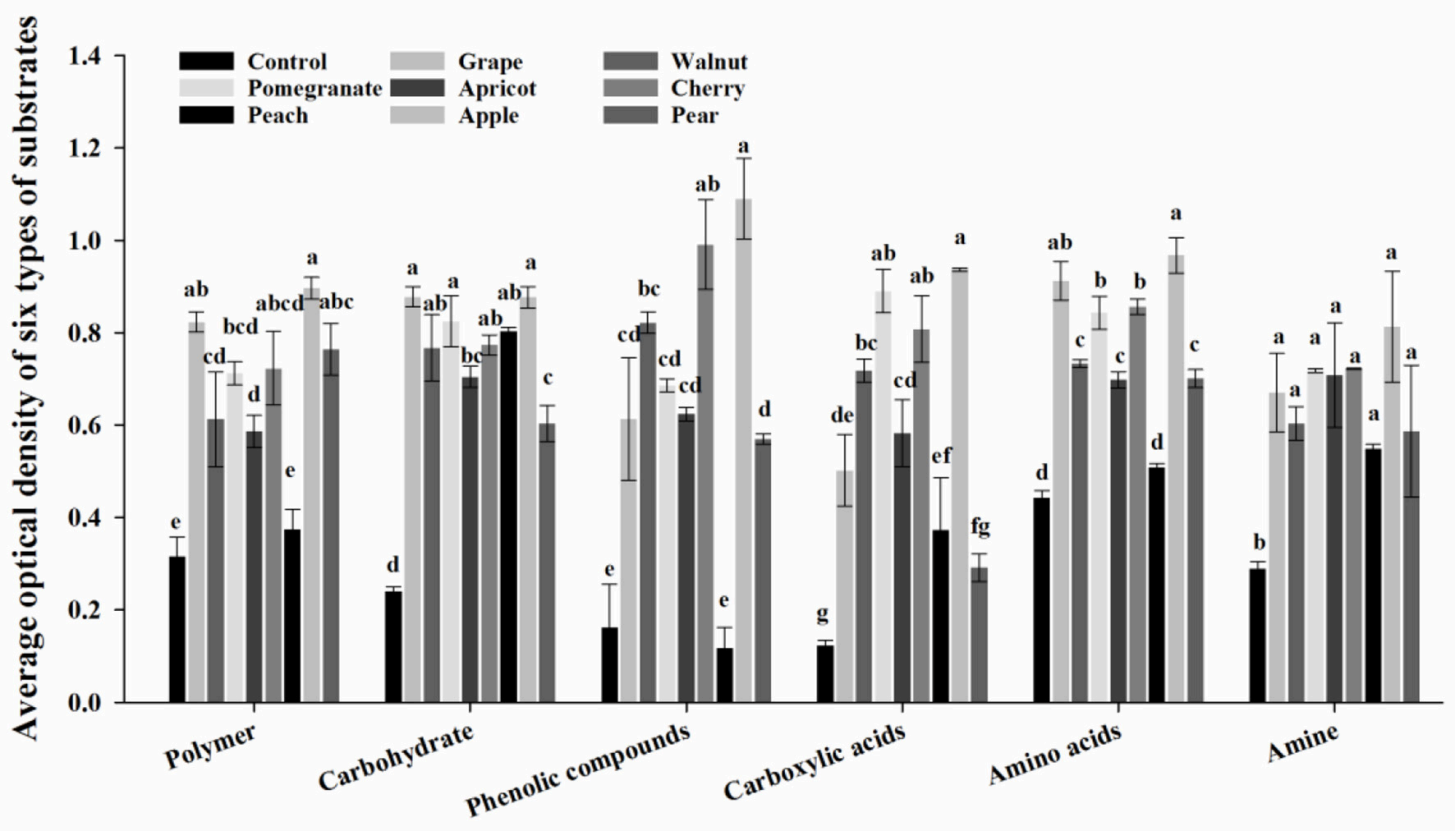
Effect of different deciduous fruit trees on the rhizosphere microbial community utilization of six types of carbon substrates at 96 h. Values are means ± SE, and letters denote significant differences among trees using one-way analysis of variance with tree species as the factor.

The use of six substrate types (polymers, carbohydrates, phenolic compounds, carboxylic acids, amino acids, and amines) by deciduous fruit trees is shown in Figure 2. The use of these six substrates differed markedly among the deciduous fruit trees. Compared to the control, all six substrates were used more by the rhizosphere microbial communities. Moreover, apple had a positive effect on the utilization of substrates. Compared to other fruit trees, the utilization of the phenolic compounds, carboxylic acids, amino acids, and amines was reduced in pears and peaches. Meanwhile, there was no significant difference in the utilization of substrates between walnuts and apricots.

### Rhizosphere Soil Microbial Community Composition

The effects of the various deciduous fruit trees on the richness and diversity of bacteria and fungi are shown in Table 3. In general, the fungal communities showed significantly lower diversity than the bacterial communities, as measured by the Chao1 index, the Shannon index and the ACE index. The rhizosphere soil bacterial and fungal diversity indices were most extreme in the apple, apricot, and walnut rhizosphere. The Chao1, Shannon, and ACE indices of bacterial diversity in the apple rhizosphere was significantly higher than others, while they were significantly lower in apricot and walnut rhizosphere. The indices of fungal diversity were significantly lower in the apple soil, while they were elevated in the walnut soil. In the apricot soil, bacterial and fungal diversity indices were quite low.

**Table 3:** Bacterial and fungal a-diversity indexes of different deciduous fruit trees.

**Microbial community**	**Treats**	**Coverage** **(%)**	**Chao1**	**Shannon**	**ACE**
Bacterial	CK	0.997 ± 0*a*	1366.865 ± 8.541*c*	8.975 ± 0.023*ab*	1342.603 ± 5.988*c*
	Grape	0.997 ± 0*a*	1397.477 ± 6.71*b*	8.95 ± 0.003*abc*	1374.639 ± 8.014*b*
	Walnut	0.996 ± 0*b*	1338.359 ± 5.659*de*	8.792 ± 0.007*d*	1319.24 ± 8.625*d*
	Pomegranate	0.997 ± 0*a*	1357.428 ± 6.295*cd*	8.885 ± 0.023*c*	1347.826 ± 2.315*c*
	Apricot	0.997 ± 0*a*	1328.005 ± 3.171*e*	8.797 ± 0.025*d*	1308.915 ± 2.401*d*
	Cherry	0.997 ± 0*a*	1368.154 ± 9.869*c*	8.891 ± 0.021*bc*	1347.091 ± 10.192*c*
	Peach	0.997 ± 0*a*	1340.351 ± 8.061*de*	8.647 ± 0.055*e*	1311.949 ± 2.072*d*
	Apple	0.997 ± 0*a*	1423.78 ± 12.723*a*	9.009 ± 0.013*a*	1408.762 ± 7.574*a*
	Pear	0.996 ± 0*b*	1365.724 ± 4.927*c*	8.75 ± 0.04*d*	1346.985 ± 7.275*c*
Fungal	CK	0.999 ± 0*ab*	466.977 ± 11.061*bc*	5.51 ± 0.482*ab*	461.813 ± 10.701*bcd*
	Grape	0.998 ± 0*c*	432.969 ± 6.426*c*	4.294 ± 0.235*b*	425.554 ± 10.345*cd*
	Walnut	0.999 ± 0*bc*	561.148 ± 13.228*a*	6.082 ± 0.041*a*	556.862 ± 12.517*a*
	Pomegranate	1 ± 0*a*	475.054 ± 26.096*bc*	6.311 ± 0.088*a*	467.928 ± 21.481*bc*
	Apricot	0.999 ± 0*bc*	332.629 ± 33.117*d*	2.095 ± 0.747*c*	334.31 ± 33.561*e*
	Cherry	0.999 ± 0*ab*	416.18 ± 13.587*c*	5.473 ± 0.158*b*	409.491 ± 10.173*cd*
	Peach	0.999 ± 0*bc*	529.852 ± 34.813*ab*	6.533 ± 0.047*a*	519.31 ± 25.267*ab*
	Apple	0.998 ± 0*c*	399.241 ± 36.439*cd*	1.864 ± 0.644*c*	398.894 ± 28.223*d*
	Pear	0.999 ± 0*ab*	447.645 ± 11.559*c*	6.05 ± 0.568*a*	442.215 ± 12.556*cd*

*Values are means ± SE. Suffixes in the same row indicate statistical significance at p < 0.05 using one-way analysis of variance with tree species as the factor*.

**Figure 3:**
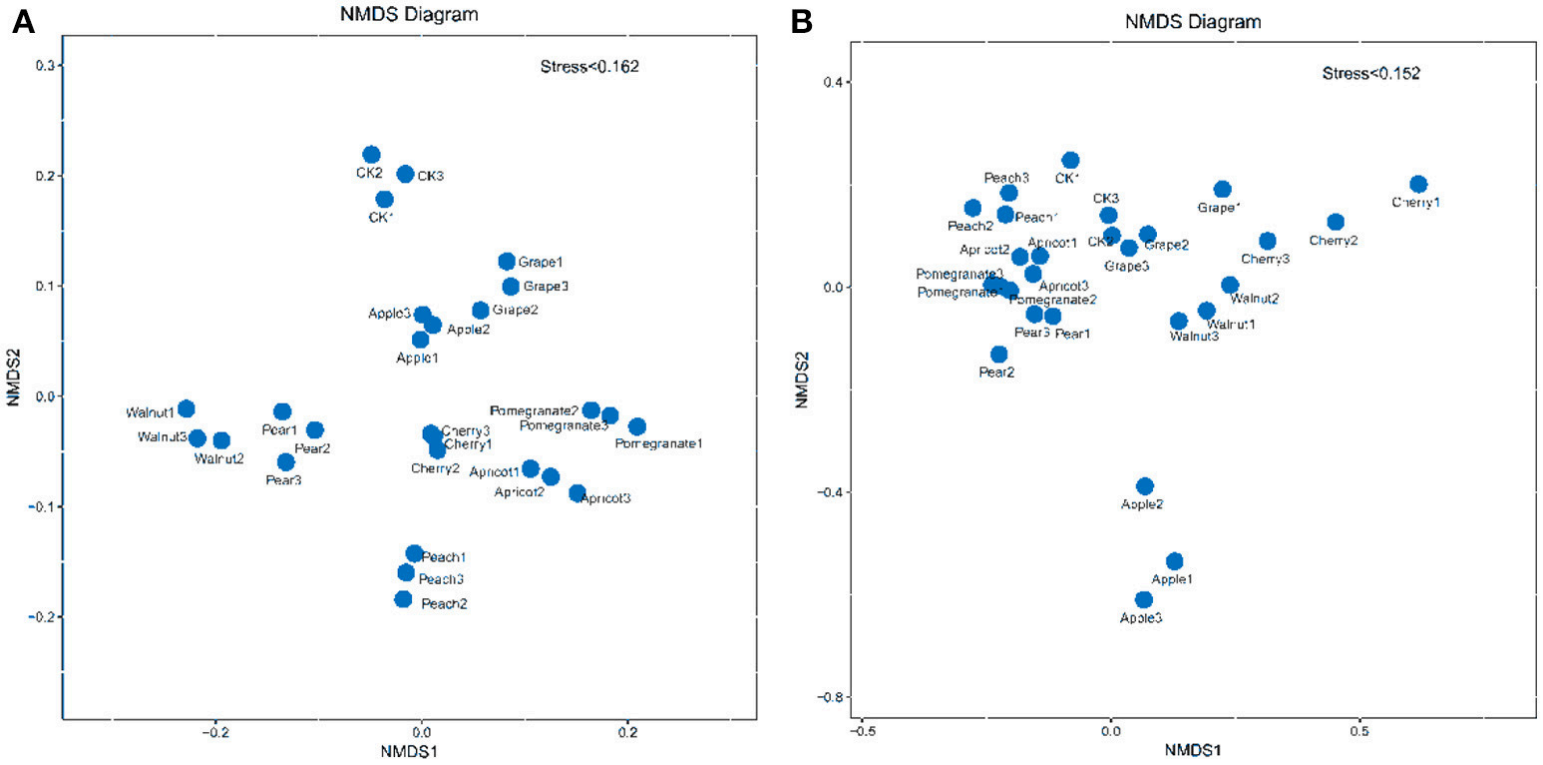
Non-metric multidimensional scaling (NMDS) plots of bacterial **(A)** and fungal communities **(B)**.

To visualize the differences in rhizosphere soil microbial community structure among the fruit tree species, taxonomic abundance profiling was performed to compute a Bray–Curtis similarity matrix divided into two dimensions using non-metric multidimensional scaling (NMDS) (Figure 3). NMDS analysis of the bacterial community revealed that each fruit tree species formed its own cluster. In contrast, the fungal samples were grouped into two clear clusters, with the apple group below and the other samples above. Furthermore, the fungal communities of the pomegranate, pear, and apricot soils were more similar to each other compared to the other tree species. Therefore, differences in rhizosphere soil microbial communities between the control and deciduous fruit trees lay mainly in the composition of the bacterial community, while the fungal community showed no significant differences. In addition, the apple tree played an important role in developing the fungal community and shaping rhizosphere microbial communities.

**Figure 4:**
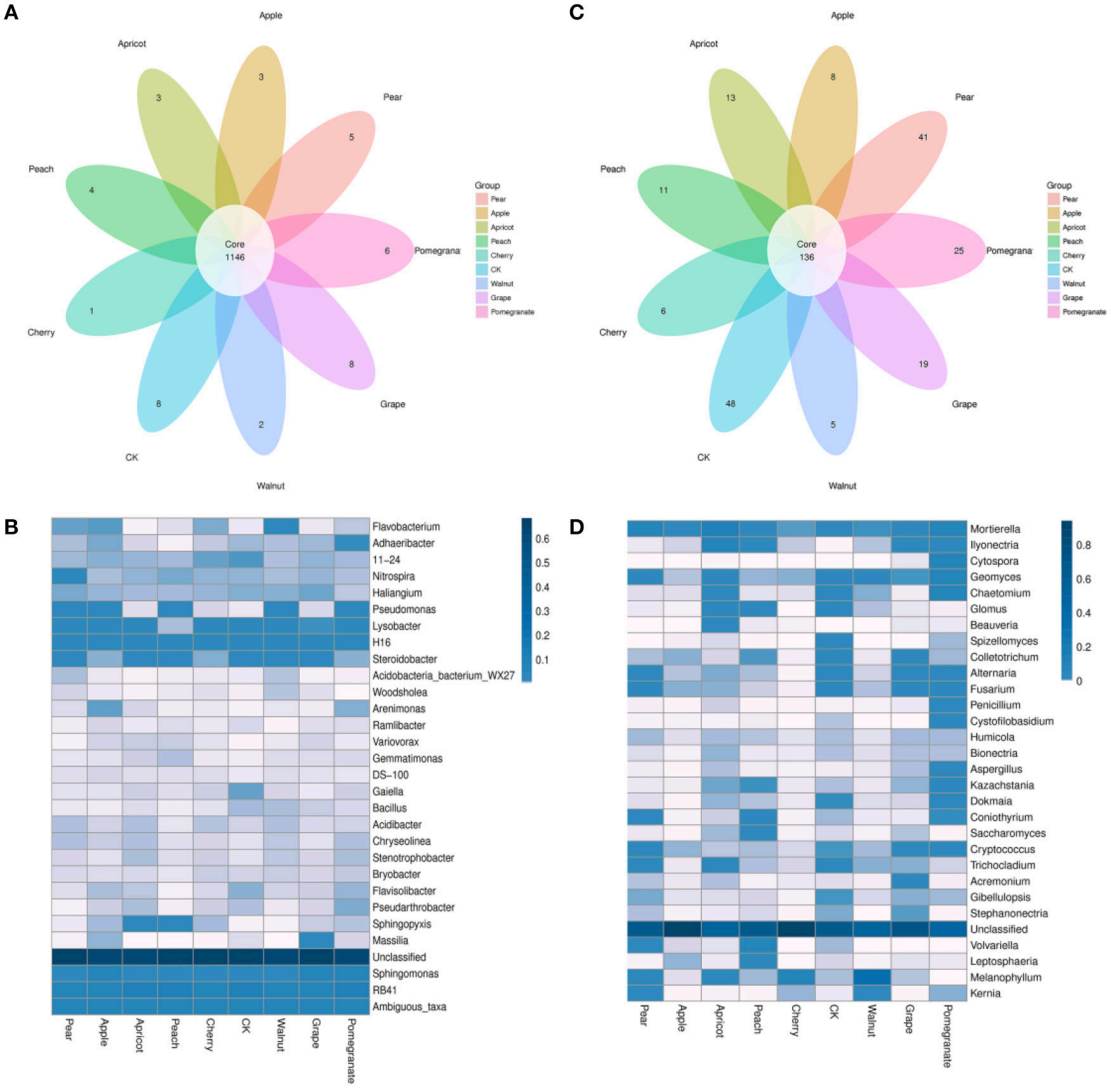
Comparison of operational taxonomic units (OTUs) in the rhizosphere bacterial **(A,B)** and fungal **(C,D)** communities of different deciduous fruit trees. The Venn diagram depicts OTUs (97% sequence identity) that are shared or unique for different soil samples **(A,C)**. The heatmap illustrates the relative abundance of the top 30 bacterial and fungal genera **(B,D)**, and the scale bar shows the variation range of the normalized abundance of the bacterial community, indicating the differences in data among these trees. The left names are genera, and the color value of each square for each row indicates the relative abundance of genera.

The shared OTUs among the eight species of deciduous fruit trees and the control sample were illustrated using a Venn diagram (Figure 4). In the bacterial communities, a total of 1,146 OTUs were shared by the nine samples, accounting for 73.51% of the 1,559 total OTUs obtained (Figure 4A). Some OTUs were only found in a particular bacterial community, accounting for two, three, five, eight, and six bacterial taxa in the walnut, apple, pear, pomegranate, and grape soils, respectively. Thus, 0.13, 0.19, 0.32, 0.38, and 0.51% of the detected OTUs were unique to the walnut, apple, pear, pomegranate, and grape soils, respectively. In addition, one, four, and three OTUs (0.06, 0.26, and 0.19%) were detected only in the cherry, peach, and apricot soils, respectively. In summary, each deciduous fruit tree had its own unique bacterial taxa.

In the fungal communities, a total of 136 OTUs were shared by the nine communities, accounting for 11.8% of the 1,148 total OTUs obtained (Figure 4C). Similar to the bacterial communities, each species of deciduous fruit tree had its own unique fungal taxa. The number of unique fungal taxa was greater in the control and pear soils compared to the other soil samples, corresponding to 4.18 and 3.57% of the OTUs detected, respectively. In addition, 25, 19, 13, and 11 OTUs (2.18, 1.66, 1.13, and 0.96%) were only detected in the pomegranate, grape, apricot and peach rhizospheres, respectively. The Venn diagram revealed that significantly fewer core taxa were shared by all nine communities, and more unique taxa were present in the fungal communities.

A heatmap of bacterial and fungal community composition based on the relative abundances of genera in the rhizospheres of various deciduous fruit trees are shown in Figures 4B,D. The microbial communities showed noticeable and significant differences among the rhizospheres of various deciduous fruit tree species. In the bacterial communities, *RB41, Ambiguous_taxa, Sphingomonas*, and *H16* had high relative abundances in all communities, and there was no significant difference among communities. However, the distribution of other bacterial genera in the nine communities varied significantly. Compared to bacterial communities, the fungal communities showed more significant differences among the nine samples.

**Figure 5:**
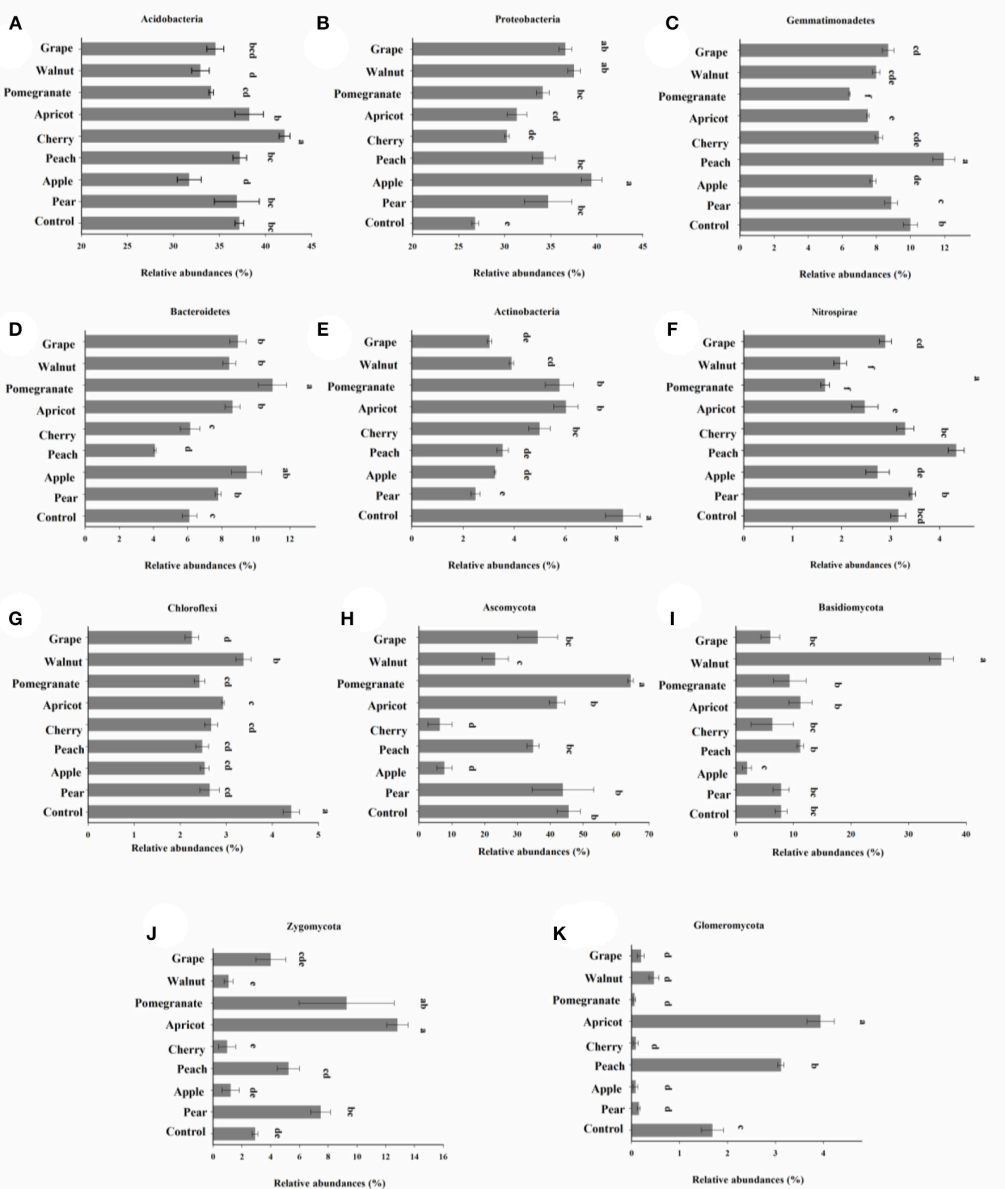
Relative abundances (%) of bacterial phyla (**A–G**; >1.0% of total composition in all samples) and fungal phyla (**H–K**; >0.01% of total composition in all sample). Values are means ± SE. Suffixes in the same row indicate statistical significance at *p* < 0.05 using one-way analysis of variance with tree species as the factor.

The bacterial OTUs were classified into 31 phyla. *Acidobacteria* was the most abundant (36.08% of all sequences), followed by *Proteobacteria* (33.89%), *Gemmatimonadetes* (8.6%), and *Bacteroidetes* (7.85%) across our dataset. Phylogenetic characterization of 16S rRNA gene amplicon sequencing data at lower taxonomic levels revealed significant differences in the number of phyla present among fruit tree species (Figure 5). We observed greater relative abundances of *Actinobacteria* and *Nitrospirae*, and a lower relative abundance of *Proteobacteria*, in the control samples compared to the deciduous fruit tree samples (Figures 5A,B,F). Among the eight kinds of fruit trees, the relative abundance of *Acidobacteria* was greatest and that of *Proteobacteria* was lowest in the cherry soil. In addition, the relative abundance of *Bacteroidetes* was greater, and the relative abundances of *Gemmatimonadetes* and *Nitrospirae* were lower, in the pomegranate soil than in the soils from other trees.

Fungal OTUs were classified into 12 phyla. The deciduous fruit tree soils were dominated by *Ascomycota* (32.33% of sequences) and *Basidiomycota* (11.21%), with OTUs assigned to *Zygomycota* (5.26%) and *Glomeromycota* (1.01%) also present. Similar to the relative abundances of bacterial phyla, the fungal phyla in the soil differed notably among the eight species of deciduous fruit trees. In addition, the relative abundances of fungi in the control and fruit tree soils did not differ significantly. Among the tree species, the relative abundances of four fungal phyla in the cherry rhizosphere were all low, especially *Ascomycota* and *Zygomycota*. Compared to other fruits, *Ascomycota* (excluding cherry) and *Basidiomycota* had low relative abundances in the apple soil. *Ascomycota* and *Basidiomycot*a were significantly higher in abundance in the pomegranate and walnut soils, respectively, compared to the other tree species. Furthermore, the relative abundances of *Zygomycota* and *Glomeromycota* were highest in the apricot soil.

To clarify differences in the rhizosphere microenvironment among the eight deciduous fruit trees, PCA analysis (Figure S1) of soil parameters was performed. The results showed that each fruit tree species has its own cluster, indicating that soil available nutrient content, soil enzyme activity and major microbial genera were significantly affected by tree species. Walnut samples clustered far away from other samples, indicating that the rhizosphere microenvironment of walnut trees differed markedly from that of the others. The apricot, cherry, and peach clusters were close, as were the apple and pear clusters. Interestingly, apricots, cherries, and peaches belong to *Prunoideae*, while apples and pears belong to *Maloideae*. This finding suggested that the rhizosphere microenvironment might be related to the subfamily of fruit trees among these deciduous fruit tree species.

### Relationships Between Soil Microbial Communities and Environmental Factors

**Figure 6:**
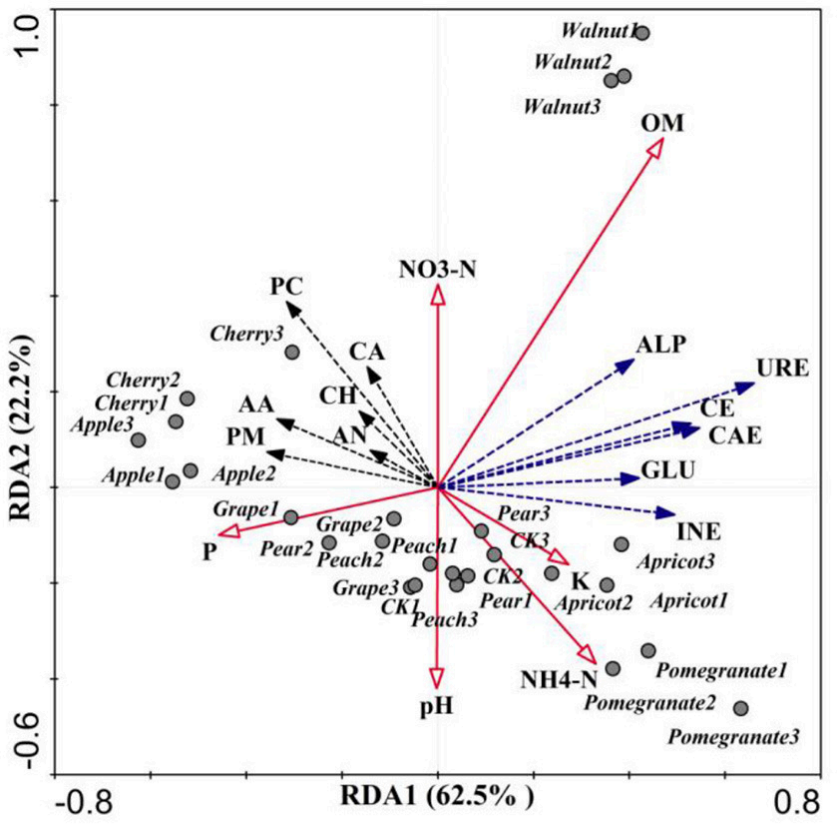
Redundancy analysis (RDA) of microbial communities using bacterial and fungal genus relative abundances. Community-level physiological profile (URE, urease; CAE, catalase; GLU, β-glucosidase; CE, cellulase; INE, invertase; ALP, alkaline phosphatase) of six types of substrates (PM, polymers; CH, carbohydrate; PC, phenolic compounds; CA, carboxylic acids; AA, amino acids; AN, amines) and mineral element content (OM, organic matter; K, available potassium; P, available phosphorus).

Redundancy analysis (RDA; Figure 6) was conducted to investigate the relationships among environmental factors, bacterial communities, CLPP, and soil enzymes. RDA1 and RDA2 accounted for 84.7% of the total variation (Figure 6). These four axes were considered significant variables and together explained 90.9% of the total variation, of which the first and second axis explained 62.5 and 22.2%, respectively. RDA analysis showed that ammonia nitrogen content, soil pH and invertase activity were closely related to the soil microbial community. The Pearson correlation coefficients between relative abundances of bacterial and fungal genera and environmental factors showed that the soil microbial utilization of the six C sources, nitrate nitrogen content, and invertase activity were negatively correlated with *Ambiguous* and *Alternaria*, but positively correlated with pH (Table 4). The ammonia nitrogen content was positively correlated with C source utilization and negatively correlated with *Ambiguous, Lysobacter, Nitrospira, Alternaria, Fusarium*, and *Colletotrichum* abundance. Invertase activity was positively correlated with CH, CA, AA, AN, and OM content. Interestingly, invertase activity was closely related to the fungal community, exhibiting positive correlations with *Mortierella, Geomyces, Lysobacter*, and *Chaetomium*, and negative correlations with *Alternaria, Fusarium*, and *Colletotrichum*. In addition, *Ambiguous* had the most significant negative correlation with microbial C utilization.

**Table 4:** Pearson correlation coefficients between microbial genus relative abundances and environmental factors.

		**PM**	**CH**	**PC**	**CA**	**AA**	**AN**	**OM**	**pH**	**K**	**P**	**NN**	**ANN**	**URE**	**CAE**	**GLU**	**CE**	**INE**
*Ambiguous*	−0.510^**^	−0.747^**^	−0.549^**^	−0.819^**^	−0.718^**^	−0.586^**^	−0.138	0.691^**^	−0.14	0.193	−0.052	−0.628^**^	−0.1	−0.261	−0.212	0.007	−0.713^**^	−0.405^*^
*Sphingomonas*	0.577^**^	0.391^*^	0.352	0.372	0.666^**^	0.367	−0.208	−0.372	−0.182	0.193	−0.131	0.197	0.158	−0.044	−0.361	0.338	0.162	0.01
*Lysobacter*	0.472^*^	0.251	0.571^**^	0.612^**^	0.557^**^	0.414^*^	−0.147	−0.369	−0.005	−0.426^*^	−0.134	0.602^**^	0.022	−0.295	−0.209	−0.18	0.274	0.234
*Steroidobacter*	−0.493^**^	−0.069	−0.494^**^	−0.487^**^	−0.526^**^	−0.216	0.187	0.239	0.281	−0.175	0.204	−0.176	0.015	0.1	0.457^*^	0.148	−0.171	−0.034
*Pseudomonas*	0.097	0.238	0.291	0.336	0.188	0.171	0.237	−0.438^*^	0.302	−0.411^*^	0.113	0.574^**^	0.084	−0.207	−0.075	−0.012	0.044	0.256
*Nitrospira*	−0.099	−0.262	−0.298	−0.560^**^	−0.355	−0.185	−0.057	0.458^*^	.390^*^	0.169	0.632^**^	−0.496^**^	−0.102	−0.373	−0.094	−0.273	−0.388^*^	0.099
*Melanophyllum*	−0.058	0.098	0.243	0.185	0.009	0.032	0.859^**^	−0.381	−0.043	−0.272	−0.207	0.386^*^	0.449^*^	0.337	0.211	0.305	0.159	0.427^*^
*Mortierella*	−0.063	−0.044	−0.224	−0.135	−0.164	0.072	−0.016	0.312	0.283	−0.276	0.484^*^	−0.246	0.213	0.234	0.506^**^	−0.068	0.446^*^	0.432^*^
*Geomyces*	0.04	0.029	0.041	0.235	0.09	0.16	0.271	−0.132	0.302	−0.384^*^	0.465^*^	0.092	0.590^**^	0.476^*^	0.302	0.251	0.753^**^	0.589^**^
*Ilyonectria*	−0.265	0.163	−0.242	0.012	−0.187	0.086	−0.007	0.179	0.184	−0.353	0.186	0.004	−0.047	0.313	0.825^**^	−0.155	0.452^*^	0.238
*Chaetomium*	0.013	0.08	0.017	0.321	0.101	0.154	0.084	−0.175	0.184	−0.397^*^	0.269	0.17	0.430^*^	0.524^**^	0.313	0.235	0.786^**^	0.36
*Alternaria*	−0.302	−0.634^**^	−0.493^**^	−0.647^**^	−0.368	−0.438^*^	−0.428^*^	0.564^**^	−0.299	0.34	−0.035	−0.618^**^	0.137	−0.211	−0.492^**^	0.389^*^	−0.475^*^	−0.487^*^
*Fusarium*	−0.099	−0.466^*^	−0.327	−0.509^**^	−0.164	−0.38	−0.422^*^	0.405^*^	−0.305	0.475^*^	−0.015	−0.632^**^	0.222	−0.107	−0.569^**^	.515^**^	−0.392^*^	−0.433^*^
*Colletotrichum*	−0.108	−0.255	−0.403^*^	−0.3	−0.081	−0.202	−0.368	0.197	−0.35	0.448^*^	−0.289	−0.385^*^	0.009	−0.04	−0.356	0.341	−0.383^*^	−0.537^**^

Enzyme activities (URE, Urease; CAE, Catalase; GLU, β-glucosidase; CE, Cellulase; INE, Invertase; ALP, Alkaline phosphatase), six types of substrates (PM, polymers; CH, carbohydrate; PC, phenolic compounds; CA, carboxylic acids; AA, amino acids; AN, amines), mineral element content (OM, organic matter; K, available K; P, available P; NN, NO3-N; ANN, NH4-N).

** Correlation is significant at the 0.01 level.

* *Correlation is significant at the 0.05 level*.

## Discussion

The rhizosphere is essential not only for plant nutrition, health, and quality, but also for microbe-driven C sequestration, ecosystem function and nutrient cycling in terrestrial ecosystems (Berg and Smalla, 2009). In particular, for fruit trees, the biotic and abiotic characteristics of rhizosphere soil determine fruit quality and yield. Analysis of the physicochemical properties of rhizosphere soil along with the enzyme activities and microbial communities of soils among different species of deciduous fruit trees provides significant benefit for elucidating differences in the rhizosphere environments of various fruit trees, and may facilitate increased fruit production and sustainable agricultural development.

### Effects of Tree Species on Soil Physicochemical Properties and Enzyme Activities

The various tree species tested in this study had significant impacts on soil physicochemical properties, soil enzyme activity and substrate quality. Ding et al. (2011) revealed that soil pH, a factor that affects crop growth, not only influenced various soil enzyme activities, but also significantly affected the effectiveness of the substrate. RDA analysis showed that soil pH was negatively correlated with the rhizosphere microbial community's utilization of six C substrates, as well as the OM content and ammonia nitrogen content (Figure 6). Rousk et al. (2010) proposed that soil microbial communities were related to the soil pH, and found that bacterial communities are more sensitive to pH than fungal communities. In addition, rhizosphere soil pH decreased significantly with the planting of fruit trees (Table 1), which can be attributed to the large amount of organic acids secreted by fruit roots. Augusto et al. also found that tree species promoted greater soil acidification and decreased pH in European forests (Augusto et al., 2002). Interestingly, the secretion of organic acids not only decreases the pH of the rhizosphere soil, but can also recruit microorganisms, leading to changes in soil microbial communities and the formation of OM and ammonia nitrogen (Haichar et al., 2008; Dennis et al., 2010). Because soil pH is a critical parameter that influences the bioavailability of many toxic and nutritive elements, as well as the physiology of the roots and the rhizosphere microbial community, rooted-mediated changes in pH are of ecological importance (Hinsinger et al., 2003). Moreover, the rhizosphere is dynamic and active, with newly generated C, derived from root secretions, and ancient C in SOM both available for microbial growth (Haichar et al., 2008).

Tree species have multiple effects on rhizosphere soil through many mechanisms, including the rates of nutrient input, output, and cycling (Augusto et al., 2002; Knops et al., 2002). Given the gradual increase of the area planted with fruit trees, it is now essential to clarify soil nutrient cycling in various species of fruit trees. In terms of soil biology and ecology, understanding available nutrients in soil and their interactions with rhizospheric processes is an important subject. The contents of soil nutrients (except for available P) in the rhizosphere increased in the presence of fruit trees in this study (Table 1). A study on plant–soil interactions by Ehrenfeld (2003) showed that the introduction of a new plant species has the potential to alter many components of the water, C, N, and other cycles in an ecosystem, ultimately resulting in an increase in the available mineral content of the rhizosphere soil. In addition, the mineral contents of the rhizospheres of different fruit trees differed significantly. This effect may be related to fruit tree species and the size of the fruit tree. Weand et al. (2010) revealed that N retention and soil C storage in a forest are influenced by the associated soil microbes and tree species. Further, microbial community structure and function data at the tree-species level will be required for prediction of long-term C and N dynamics (Weand et al., 2010). Moreover, evidence from Knops et al. (2002) showed that plant species could strongly influence ecosystem N cycling. Therefore, the effect of tree species is exerted via impacts on N inputs and losses, rather than solely by differences in plant quantity and quality. Soil N is closely related to OM. Furthermore, root exudates such as citric acid and malic acid can increase soil nutrient levels and improve the soil environment (Hou et al., 2018). Additionally, plant species have large impacts on the composition and activity of the microbial communities in rhizospheric soils, and this influence has the potential to cause differences in available nutrient contents. Our results indicated that the magnitude of the rhizosphere effect on SOM accumulation varied dramatically, from 102.6 to 201.2% of the control, and was mainly affected by tree species (Table 1). Knops et al. (2002) also supported the hypothesis that N released from SOM could be incorporated by microbes. However, they found no correlation between SOM and N using RDA (Figure 1). Cheng (2009) revealed that rhizospheric enhancement of soil C mineralization in planted treatments did not result in a proportional increase in net N mineralization, suggesting a possible decoupling of C cycling and N cycling in the rhizosphere. Hence, C cycling and N cycling in the rhizosphere of eight fruit trees appeared to be decoupled. However, urease, an important enzyme involved in soil N cycling, was positively correlated with SOM, showing that the C and N cycles are coupled (Figure 5). This result may be attributed to limited N content in the soil, which leads to greater immobilization and less mineral N availability in some rhizosphere environments.

Soil enzymes, which are related to OM dynamics and mineral nutrient cycling in the soil, are considered sensitive early indicators of alterations in soil quality (Puglisi et al., 2006; Zhang et al., 2011; Paz-Ferreiro and Fu, 2016). The RDA revealed that the activities of cellulase and invertase were positively correlated with OM content (Figure 6). Alvarenga et al. (2008) found that cellulase and invertase are highly sensitive to substrate availability and are involved in the decomposition of OM and the release of reducing sugars as end products of the C cycle (Zhang et al., 2011). Phosphatases provide an excellent indicator of the organic P mineralization potential and biological activity in soils (Dick and Tabatabai, 1993; Kotroczó et al., 2014). Alkaline phosphatase activity was positively correlated with *Melanophyllum, Mortierella*, and *Geomyces*, and negatively correlated with *Alternaria, Fusarium*, and *Colletotrichum* (Table 4). Urease activity is often used as an index of N mineralization, as it is involved in catalyzing the hydrolysis of organic N to inorganic N in the soil (Sardans et al., 2008). RDA analysis revealed that urease activity was positively correlated with SOM. Similarly, Myers et al. found that urease activity is strongly correlated with the SOM content (Myers and McGarity, 1968; Dalal, 1975; Bremner and Mulvaney, 1978; Roscoe et al., 2000). Furthermore, catalases are primarily associated with soil respiration and microbial activity, and they reflect the intensity of soil microbial processes (Haichar et al., 2008; Yu et al., 2016). Catalase activity was positively correlated with *Geomyces* and *Chaetomium* abundances (Table 4). As shown in Table 2, soil enzyme activity showed significant differences among fruit tree species. Chodak and Niklinska (2010) revealed that the activity of acid phosphomonoesterase was not affected by tree species, while dehydrogenase and urease activities were significantly affected by tree species and were greatest under pine (Chodak and Niklinska, 2010). Brockett et al. (2012) found that enzyme profiles differed among forest types, and phenol oxidase and peroxidase activities differed from other enzyme activities in seven forest types (Brockett et al., 2012). Weand et al. (2010) illustrated that soil enzymes were not affected by tree species, but were significantly affected by fertilizer application.

### Effects of Tree Species on Microbial Communities

Plants can influence rhizosphere microbial communities, which are essential to crop growth and yield (Kodama et al., 2011; Berendsen et al., 2012; Sugiyama et al., 2014). In this study, the rhizosphere microbial communities of different deciduous fruit trees grown for 5 years in the field were analyzed using PCR and Illumina sequencing to identify trends in the development of microbial community composition, and using the BIOLOG EcoPlate to construct CLPPs and clarify the rhizosphere soil environments of different deciduous fruit trees.

As a rapid screening method for detecting differences among different treatments, CLPP has been widely applied to characterization of microbial communities in numerous habitats, ranging from native soil to sewage sludge-amended soil and organic compost (Doran et al., 1996; Sharma et al., 1998; Insam and Goberna, 2004; Frac et al., 2012). Furthermore, this method has the potential to overcome the disadvantages of culture-based analyses and biochemical tests, and it is frequently employed to determine the effects of various environmental factors on soil properties based on the catabolic characteristics of microbial communities (Haack et al., 1995; Schutter and Dick, 2001; Preston-Mafham et al., 2002). In this study, Biolog substrate utilization assays revealed that the metabolic capabilities of peach rhizosphere soil were lower than those of other deciduous fruit tree soils (Figure 2). However, apple rhizosphere soil had the highest microbiological activity among the deciduous fruit trees investigated. This finding indicates that, given the same soil background, the metabolism of rhizosphere microbial communities around deciduous fruit trees differs 5 years after planting, which may be related to differences in the growth of tree roots, rhizosphere secretions and fertilizer requirements. However, further verification of these hypotheses is needed.

Soil microbial communities are the largest known reservoir of biological diversity in the world and are important for plant growth (Curtis et al., 2002; Torsvik et al., 2002; Gams, 2007; Buée et al., 2010; Berendsen et al., 2012). The rhizosphere microbiome, which is shaped by root secretions, can influence plants through genetic elements and interactions (Berendsen et al., 2012). Therefore, investigating the composition of the rhizosphere microbiome and its relationship with environmental parameters is essential. In this study, bacterial α-diversity indices of apple soil were higher than those of other deciduous fruit tree soils, while those of walnut were relatively low (Table 3). Conversely, the fungal α-diversity indices of the walnut soil were higher than those of other deciduous fruit tree soils, while those of the apple soil were relatively low. Moreover, the apricot soil had relatively low bacterial and fungal α-diversity indices. Hence, significant differences were observed in rhizosphere microbial community diversity among the walnut, apple and apricot soils. Moreover, the microbial community results showed that the rhizosphere microbiome was significantly affected by tree species. Similar studies have indicated that each plant species was colonized by specific microbial populations, and that many plant disease-causing organisms, including bacteria and fungi, have co-evolved with plants and exhibit high host specificity (Long, 2001; Berg and Smalla, 2009; Raaijmakers et al., 2009; Costa et al., 2010). Costa et al. (2010) revealed that plant species was a crucial factor in shaping the differentiation of actinobacterial communities in the strawberry rhizosphere from bulk soil. Similarly, according to the Venn diagram results in this study, each tree species had its own unique OTUs. The relative abundances of bacterial and fungal phyla showed that the composition of the rhizosphere microbial communities associated with the eight deciduous fruit trees differed significantly in terms of the nine dominant bacterial and fungal phyla. Furthermore, the composition of microbial communities in the rhizosphere soil of different deciduous fruit trees revealed that fungal communities have more unique taxa and less diversity than bacterial communities. Urbanová et al. (2015) also discovered that fungal communities were less diverse than bacterial communities, and showed a greater tendency to be tree-specific in a forest ecosystem. Hence, the effect of trees species on the composition of the microbial community was found to be especially strong for fungi.

### Rhizosphere Microbes of Deciduous Fruit Trees Were Related to Soil Physicochemical Properties and Enzyme Activities

Pearson correlation coefficients between the relative abundances of bacterial and fungal genera and environmental factors showed that soil microbial utilization of the six C sources, the nitrate nitrogen content and invertase activity were negatively correlated with *Alternaria*, but positively correlated with pH (Table 4). The *Alternaria* genus includes both plant-pathogenic and saprophytic species, which may affect crops in the field or result in harvest and postharvest decay of plant products (Logrieco et al., 2009; Dean et al., 2014). This genus can produce diverse secondary metabolites, including toxins (Montemurro and Visconti, 1992), and some of these metabolites are effective mycotoxins (e.g., alternariol, alternariol methyl ether, tenuazonic acid, etc.) with mutagenic and teratogenic properties (Liu et al., 1991; Dean et al., 2014). Therefore, *Alternaria* has the potential to inhibit the growth of other microbes or organisms, resulting in decreased metabolic capacity of the microbial community and invertase activity in the soil. Furthermore, the ammonia nitrogen content was negatively correlated with *Lysobacter, Nitrospira, Alternaria, Fusarium*, and *Colletotrichum* (Table 4). *Nitrospira*, a group of nitrite-oxidizing bacteria, are the most widespread nitrifiers in the environment (Lücker et al., 2010; Koch et al., 2015). Hence, *Nitrospira* had a strong negative correlation with the ammonia nitrogen content. Members of the genus *Lysobacter* are Gram-negative bacteria widely distributed in freshwater, soil and plant environments, and the genus owes its name to its lytic effects on other microorganisms (de Bruijn et al., 2015). Daft et al. suggested that the spectacular lytic capabilities of *Lysobacter* species suggest that they might play a critical role in the control of microbial populations in nature (Daft et al., 1975; Reichenbach, 2006). In addition, *Lysobacter* can synthesize new secondary metabolites, such as phenazine-*N*-oxide and myxin, which inhibit the growth of or destroy other microorganisms (Peterson et al., 1966; Weigele and Leimgruber, 1967). Fusaric acid is a secondary metabolite produced by certain species of *Fusarium* that can cause wilt disease in tomatoes, cotton, peas, bananas, watermelons, and other crops (Wood et al., 1972; Bacon et al., 1996; Watanabe et al., 2011). Invertase activity was positively correlated with CH, CA, AA, AN, and OM content in this study. In particular, invertase was closely related to the fungal community, showing positive correlations with *Mortierella, Geomyces, Lysobacter*, and *Chaetomium*, and negative correlations with *Alternaria, Fusarium* and *Colletotrichum*. Interestingly, Franke demonstrated that the genus *Mortierella* was the most abundant fungal genus showing a strongly positive correlation with plant growth (Franke-Whittle et al., 2015). Members of the genus *Geomyces* can form mycorrhizae with the roots of plants, such as alpine *Ericales*, allowing these plants to adapt to low-nutrient environments (Dalpé et al., 1989). Members of the genus *Chaetomium* are considered a rich source of novel and bioactive secondary metabolites, with highly promising potential applications (Li et al., 2014), and most of these fungal metabolites exhibit antitumor, cytotoxic, antimalarial, enzyme-inhibitory, antibiotic activities, among others (Zhang et al., 2012). The genus *Colletotrichum*, the eighth most important phytopathogenic fungus in the world, is a large genus of *Ascomycete* fungi containing many species that cause anthracnose or blight in a wide range of important crop and ornamental plants (Bailey and Jeger, 1992; Perfect et al., 1999; Dean et al., 2012). Interestingly, in this study, *Mortierella, Geomyces, Lysobacter*, and *Chaetomium* were plant-beneficial microbes, while *Alternaria, Fusarium*, and *Colletotrichum* were categorized as plant pathogens. These results showed that invertase activity was not only closely related to the metabolic activities of the rhizosphere microbial community, but also positively correlated with plant-beneficial microbes such as *Mortierella*. Invertase activity was negatively correlated with pathogenic microbes such as *Fusarium*, among the main fungal genera observed. Therefore, soil invertase activity, an important indicator of soil quality, is closely related to many soil parameters and has the potential to indicate the rhizosphere soil state of deciduous fruit trees in northern China.

### Further Research: Rhizosphere Microbes Secrete Soil Enzymes to Reach Their Catalytic Potential in the Rhizosphere

The catalytic potential of rhizosphere microbes is mainly attained through secretion of various soil enzymes involved in soil nutrient cycling and resource exchange (Aon and Colaneri, 2001; Cardon and Gage, 2006; Robertson and Groffman, 2007; Mohammadi, 2012). In this study, six soil enzymes were chosen to clarify the relationship between soil enzyme activity, available nutrient content, and the dominant genera of soil microbes. Our results indicated that invertase and urease are closely related to soil available nutrient content and major microbial genera (Table 4 and Table S4), and thus may play a major role in the rhizosphere soil environment. To further elucidate the catalytic potential of microbes in the rhizosphere microenvironment, research on processes such as N fixation, phosphate solubilization, and metal quenching potential will be conducted in the future.

## Conclusion

This study demonstrated that fruit tree species had significant impacts on soil physicochemical properties, soil enzyme activities, and the soil microbial community, suggesting that the microenvironment of the rhizosphere soil was shaped by the presence of eight common deciduous fruit trees and was closely related to soil microbes in northern China. The correlations among rhizosphere parameters showed that pH was negatively correlated with the utilization of the six C sources in the rhizosphere soil ecosystem of the deciduous fruit trees, indicating that soil pH has the ability to affect the metabolism of microbial communities. Correlations between the soil ammonium nitrogen content and various indicators were also observed, indicating that the sensitivity of soil ammonium nitrogen to environmental changes may allow it to be used as an indicator. The SOM and *Geomyces* were positively correlated with urease, catalase, invertase and alkaline phosphatase activities, revealing that OM and *Geomyces* may have close relationships with soil enzymes, and could have the potential to enhance soil enzyme activity. Invertase activity was not only closely related to the metabolic activities of the rhizosphere microbial community, but was also positively related to plant-beneficial microbes such as *Mortierella* and negatively correlated with pathogenic microbes such as *Fusarium*, among the main fungal genera.

## Author Contributions

PS, WS, and GW contributed to the conceptualization of the study. WS contributed to the data curation and formal analysis. ZW and GW contributed to the funding acquisition. WS, HY, and XY contributed to the investigation. WS and XY contributed to the methodology. PS and WS contributed to the project administration and wrote the original draft of the manuscript. PS and GW contributed to the resources. WS and HY contributed to the software. PS, WS, HY, XY, DG, XQ, ZW, and GW contributed to the supervision and visualization. PS and GW contributed to the validation. WS, PS, and HY wrote, reviewed and edited the manuscript.

### Conflict of Interest Statement

The authors declare that the research was conducted in the absence of any commercial or financial relationships that could be construed as a potential conflict of interest.

## Supplementary Material

Table S1 Sequencing data details of bacterial communities.

Table S2 Sequencing data details of fungal communities.

Table S3 Rhizosphere soil trace element content of different deciduous fruit trees.

Table S4 Pearson correlation coefficients between of soil enzymes and environmental factors.

Figure S1 Principal component analysis (PCA) of rhizosphere soil parameters (including soil available nutrient content, soil enzyme activity, and major microbial genus), showing differences among eight deciduous fruit trees.
