# Supplementary figures and images for: Rhizosphere Microenvironments of Eight Common Deciduous Fruit Trees Were Shaped by Microbes in Northern China
Source: Front Microbiol. 2018 Dec 18;9:3147. doi: 10.3389/fmicb.2018.03147 (PMC6305578; doi:10.3389/fmicb.2018.03147)

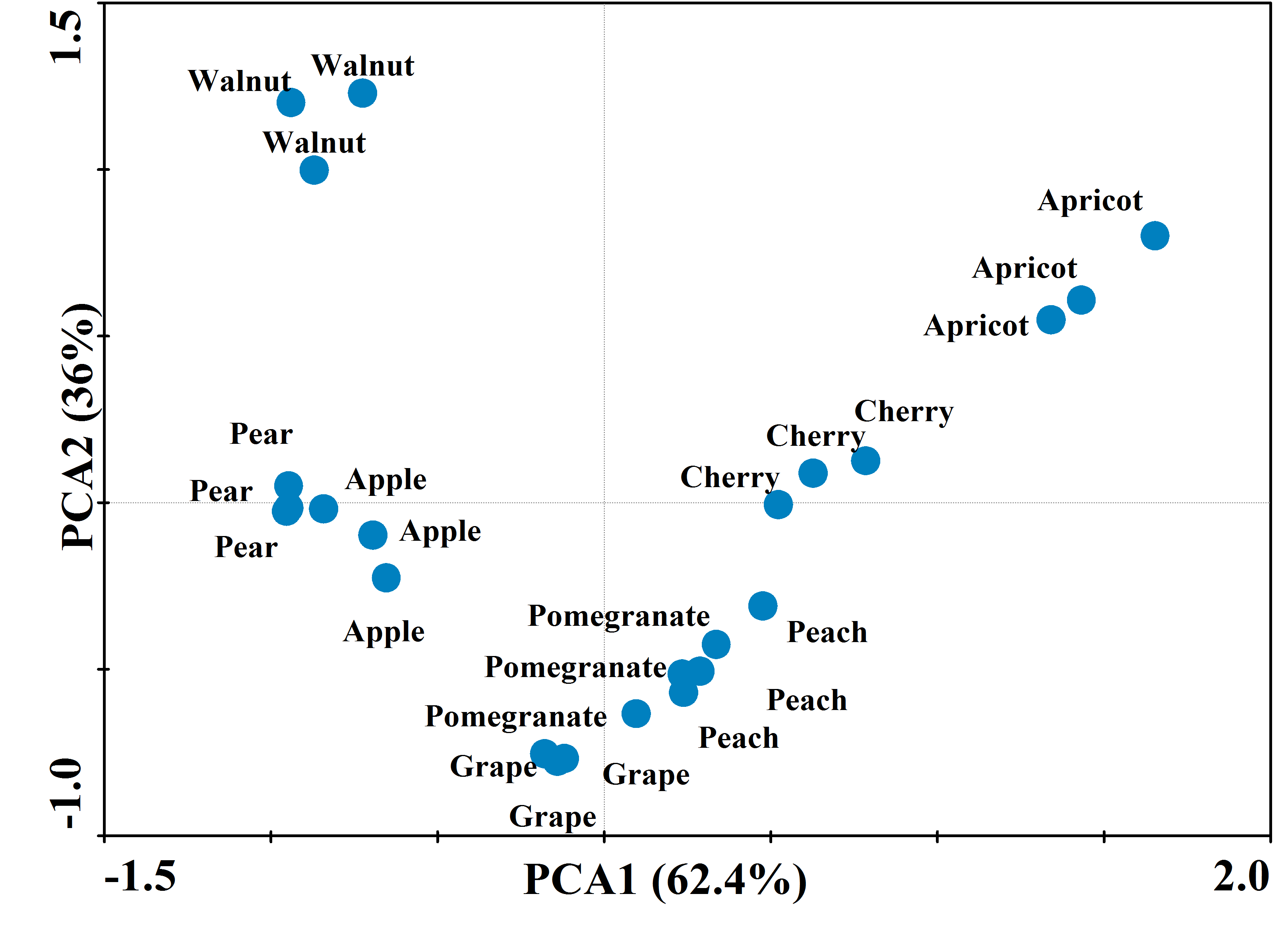

Supplement: Figure S1 — Principal component analysis (PCA) of rhizosphere soil parameters (including soil available nutrient content, soil enzyme activity, and major microbial genus), showing differences among eight deciduous fruit trees. [file Image_1.tif]
